# *strandCet*: R package for estimating natural and non-natural mortality-at-age of cetaceans from age-structured strandings

**DOI:** 10.7717/peerj.5768

**Published:** 2018-10-09

**Authors:** Camilo Saavedra

**Affiliations:** CO Vigo, Instituto Español de Oceanografía (IEO), Vigo, Spain

**Keywords:** Mortality-at-age, Natural mortality, Anthropogenic mortality, Bycatch, Strandings, Cetaceans

## Abstract

Mortality is one of the most important parameters for the study of population dynamics. One of the main sources of information to calculate the mortality of cetaceans arises from the observed age-structure of stranded animals. A method based on an adaptation of a Heligman-Pollard model is proposed. A freely accessible package of functions (*strandCet*) has been created to apply this method in the statistical software R. Total, natural, and anthropogenic mortality-at-age is estimated using only data of stranded cetaceans whose age is known. Bayesian melding estimation with Incremental Mixture Importance Sampling is used for fitting this model. This characteristic, which accounts for uncertainty, further eases the estimation of credible intervals. The package also includes functions to perform life tables, Siler mortality models to calculate total mortality-at-age and Leslie matrices to derive population projections. Estimated mortalities can be tested under different scenarios. Population parameters as population growth, net production or generation time can be derived from population projections. The *strandCet* R package provides a new analytical framework to assess mortality in cetacean populations and to explore the consequences of management decisions using only stranding-derived data.

## Introduction

Cetaceans are protected by several international agreements and legislation (e.g., Agreement on the Conservation of Small Cetaceans of the Baltic, North East Atlantic, Irish and North Seas—ASCOBANS; the “Habitats” Directive—European Council, 1992; European Parliament, 2008) and therefore effective management is needed to ensure their conservation. Modeling population dynamics provides a useful tool to assess the consequences of human activities and the impact on cetacean populations, and as such can support conservation management decisions. Mortality rate is one of the most important parameters for population dynamics and demographic studies (Caswell, 2001; Morris & Doak, 2002). Mortality risk varies according to the age of the individual, thus giving populations a certain age structure and therefore having a direct impact on their dynamics over time. One can distinguish between natural mortality (e.g., death due to disease, predation or starvation) and mortality caused directly by anthropogenic activities (e.g., fishery bycatch or ship strikes). For effective population management, it is necessary not only to know the total mortality-at-age, but also what fraction of it is due to natural processes and what fraction to anthropogenic processes, for example, fishery bycatch, which represents a significant threat to many cetacean species in the European Atlantic and other parts of the world (Northridge, 1991; Read, Drinker & Northridge, 2006; Young & Iudicello, 2007; Reeves, McClellan & Werner, 2013; Dolman et al., 2017; Peltier et al., 2016).

The most accessible source of information available nowadays about the population structure of most cetaceans is found in the strandings records. The study of mortality from dead cetaceans has generally relied on the use of life tables (Olesiuk, Bigg & Ellis, 1990; Barlow & Boveng, 1991; Brault & Caswell, 1993; Barlow & Clapham, 1997; Stolen & Barlow, 2003). Life tables allow the construction of theoretical populations with a corresponding abundance by age, assuming a stationary age distribution. A vector of age-specific survival or mortality rates is then estimated from this population structure (Caughley, 1966). However, mortality and survival rates directly derived from observational data are generally imprecise and may be biased (e.g., underrepresentation of young ages—predation; higher presence of individuals dead near the coast—some ages, sex or bycaught individuals; etc.), and therefore model-based estimates are used. The most common model used for such purposes is the Siler model (Siler, 1979). This model divides mortality into juvenile, constant and senescent components and fits the expected mortality patterns of long-lived species, such as marine mammals, reasonably well; as shown by different works in which it has been applied to several cetacean populations (Barlow & Boveng, 1991; Stolen & Barlow, 2003; Mannocci et al., 2012). However, the Siler model does not allow the disentangling of natural mortality from anthropogenic mortality. Secchi & Fletcher (2004) proposed an extension of the Siler model, later improved using reproductive data and adapted to a Bayesian framework by Moore & Read (2008). This method uses age-structured strandings and bycatch to study cetacean demography in a mixture model that also allows the study of natural mortality. However, this important breakthrough in the demographic study of cetaceans requires a considerable amount of data on bycatch from fisheries which is lacking for most of the fisheries in the world.

In this study, a Heligman-Pollard model (HP) (Heligman & Pollard, 1980) has been adapted to estimate total, natural and anthropogenic mortality-at-age of cetaceans using only age-structured strandings data and the causes of death. The HP follows a similar pattern as the Siler model but includes more flexibility to model an extra mortality component. This model has been used in the field of human demography when an important “accident” (the authors terminology) has occurred, which has differentially affected different segments of a population thus causing modifications in its age structure (Sharrow, 2013), for example, in the case of a war (Heligman & Pollard, 1980) or an aggressive disease (Sharrow et al., 2013). This approach can be extended to estimate mortality-at-age of a marine mammal population whose pattern of mortality behaves in a similar way when subjected to an external pressure (e.g., bycatch), which mainly affects certain ages (Moore & Read, 2008). The estimated mortality-at-age can be used to assess how the abundance and age structure of a population change over time applying a Leslie matrix (Leslie, 1945) and calculating its population parameters, which allows for the assessment of different management scenarios.

The free and open-source programming software R (R Core Team, 2017) is widely used among statisticians, data miners and other scientists (Tippmann, 2015). Several specific packages have been recently developed although only a few in the field of marine mammal science, and have been aimed at the calculation of abundance estimates (McClintock, 2015; Miller, 2016) or for handling satellite tracking data (Sumner, 2016), but none specifically to analyze strandings data and/or mortality. In this work, a set of functions have been written and compiled in a R package called *strandCet* (Saavedra, 2018) to assess mortality in cetacean populations and explore the consequences of management decisions using only stranding-derived data.

## Materials and Methods

### *strandCet* package availability

The R package *strandCet* is freely available from CRAN at https://cran.r-project.org/web/packages/strandCet/index.html. The *strandCet* source code is available on Github at http://github.com/milokmilo/strandCet. The package has been designed to provide a user-friendly method for assessing mortality-at-age from stranded cetaceans and to explore the consequences of different management scenarios. In addition to constructing life tables, the package allows the user to estimate total mortality-at-age with a Siler model; to estimate total, natural and anthropogenic mortality-at-age with an adapted HP (aHP) and to derive population projections using Leslie models. The implemented methods and some of the core functions of the *strandCet* package are described below. Specific description of functions and other documentation is available in the R help and the package manual.

### Siler model

The Siler model is a five-parameter mortality model whose inverse expression, the survivorship at a given age *l*(*x*), is expressed as the product of three competing processes (described as risks by Siler, 1979) as denoted in Eq. (1).

(1) $$l\left(x \right) = {l_j}\left(x \right) \cdot {l_{\rm{c}}}\left(x \right) \cdot {l_{\rm{s}}}\left(x \right)$$

where ${l_j} = {\rm{exp}}\left({\left({-{a_1}/{b_1}} \right)\left({1-{\rm{exp}}\left({-{b_1}x} \right)} \right)} \right)$ is an exponentially decreasing risk due to juvenile risk factors, ${l_{\rm{c}}} = {\rm{exp}}\left({-{a_2}x} \right)$ represents a constant risk experienced by all age classes, ${l_{\rm{s}}} = {\rm{exp}}\left({\left({{a_3}/{b_3}} \right)\left({1-{\rm{exp}}\left({{b_3}x} \right)} \right)} \right)$ is the exponential increasing risk due to the senescence, *x* is a given age and *a_n_* and *b_n_* are the Siler parameters.

Conversely, the total mortality at age μ(*x*), in Eq. (2), is the sum of the juvenile mortality μ_*j*_(*x*), the senescent mortality μ_*s*_(*x*) and a constant mortality affecting all age classes μ_*c*_(*x*).

(2) $${{\rm \mu }}\left(x \right) = {{\rm{\mu }}_j}\left(x \right) + {{\rm{\mu }}_{\rm{c}}}\left(x \right) + {{\rm{\mu }}_{\rm{s}}}\left(x \right)$$

This total mortality can be calculated by using the Siler parameters (*a*_1_, *b*_1_, *a*_2_, *a*_3_, *b*_3_) as denoted in Eq. (3).

(3) $${\rm{\mu }}\left(x \right) = {a_1}{\rm{exp}}\left({-{b_1}x} \right) + {a_2} + {a_3}{\rm{exp}}\left({{b_3}x} \right)$$

This expression describes the general shape of the mortality curve using five parameters that account for an initially increasing (and subsequently decreasing) risk of an individual dying at the beginning of life, a constant risk through the whole life, and an increased risk due to senescence. A function for fitting the competing-risk Siler model from aged dead animals (termed *Si.mod*), was implemented in the *strandCet* package using the Nelder & Mead (1965) optimization method implemented in the *optim* function of the *stats* package of R.

The Siler model has been used to estimate total mortality-at-age of several species of cetaceans from a representative sample of aged stranded animals (Secchi & Fletcher, 2004; Mannocci et al., 2012). However, it is only possible to calculate the natural mortality (as opposed to total mortality) in cases where there is no other external risk to the population since the natural mortality then equals the total mortality (Stolen & Barlow, 2003). If the user is only interested in estimating the total mortality this method is appropriate.

One of the problems found when using only data on stranded cetaceans is the apparent underrepresentation of the younger animals that often occurs in the samples. The youngest ages are usually under-represented because they are more vulnerable to predation, more rapidly decomposed and/or have a lower detection probability (Stolen & Barlow, 2003). If this is not taken into account it can lead to underestimates of age-specific mortality rates in the younger age classes (Mannocci et al., 2012). This has been considered when designing this package and an extra option was included in most of the package formulas to allow the removal of these biased mortality rates from the sample, fitting the model on the remaining data (i.e., “unbiased ages”), and allowing the user to later predict the mortality for all ages (to apply this option, see the Supplemental Information, the library manual or the help of the desired function in R).

### Heligman-Pollard model

The HP is an eight-parameter non-linear and parametric model, represented in Eq. (4), which uses the following equation to fit the probability of dying as a function of age.

(4) $${q_{\left( x \right)}} = {A^{{{\left( {x + B} \right)}^{\rm{C}}}}} + D*{\rm{exp}}\left[ { - E{\rm{}}{{\Big\{ {{\rm{ln}}\Big( {{x \over F}}\Big )} \Big\}}^2}} \right] + {{G{H^x}} \over {\left( {1 + G{H^x}} \right)}}$$

where *A*, *B*, and *C* represent the mortality at age one, the difference between mortality at ages 1 and 0, and the rate of decline of infant mortality respectively. *D*, *E*, and *F* parameters are involved in describing the shape of the so-called “accident hump” which is the curve representing the mortality caused by a particular “accident” in the population (e.g., bycatch, ship strikes, disease caused by an epidemic, etc.). Those parameters represent the severity of the accident, the spread and the location of the hump respectively. *G* and *H* indicate the base level of later adult mortality and the rate of increase in mortality at the later adult ages. For more detailed information about the description and flexibility of the parameters and prior selection, see Heligman & Pollard (1980), Dellaportas, Smith & Stavropoulos (2001) and Sharrow et al. (2013).

The HP model can be decomposed into a three-term expression, each one representing a distinct component of mortality: a young mortality curve ${A^{{{\left({x + B} \right)}^{\rm{C}}}}}$, an “accident hump” in early adult life $D{\rm{\,*\,exp}}\left[ { - E{\rm{}}{{\left\{ {{\rm{ln}}\left( {x/F} \right)} \right\}}^2}} \right]$ and an adult mortality curve ${\rm{}}G{H^x}/\left( {1 + G{H^x}} \right)$. The general model shape is represented by a rapidly declining negative exponential curve in the younger ages and by a Gompertz positive exponential curve (Gompertz, 1825) which rises at the adult ages. These young and adult mortality curves are quite similar to the Siler model although slightly more flexible. The main difference between them lies in the second component, which in the HP model reflects a “hump” instead of a constant (i.e., mortality that mainly affects certain ages and thus modifies the normal mortality pattern of the population).

The parameters involved in the shape of the “accident hump” do not provide enough flexibility for the purpose of this analysis, since the beginning of this mortality curve must match the zero on the *y*-axis. This would be unrealistic in some cases, for example in cetacean bycatch, since it assumes that there is no bycatch at age 0. In order to mitigate this limitation, a ninth parameter (*I*) was added to the second component of the model. The functionality of this extra parameter is to control the height at which this curve intersects the *y*-axis. Thus, the aHP model that was used resulted as denoted in Eq. (5).

(5) $${q_{\left( x \right)}} = {A^{{{\left( {x + B} \right)}^{\rm{C}}}}} + \left\{ {I + D*{\rm{exp}}\left[ { - E{\rm{}}{{\left\{ {{\rm{ln}}\left( {{x \over F}} \right)} \right\}}^2}} \right]} \right\} + {{G{H^x}} \over {\left( {1 + G{H^x}} \right)}}$$

Due to its complexity, it is difficult to fit the HP model using standard procedures such as least squares due to the over-parameterization, and it is therefore reasonable to use a Bayesian framework to reduce such a drawback (Dellaportas, Smith & Stavropoulos, 2001; Clark, Thomas & Bao, 2012; Sharrow et al., 2013), and thus avoid, as far as possible, unrealistic results that may arise. Sharrow (2012) developed a method for fitting this model using a Bayesian Melding approach (see Poole & Raftery, 2000). Bayesian melding is designed for problems in which a deterministic model is used in the likelihood function, as is the case. The prior density for the model inputs and likelihood for the outputs and the data are combined via logarithmic pooling to produce the posterior distribution for the model inputs. A set of parameter values is sampled from the posterior distribution using an Incremental Mixture Importance Sampling (IMIS) adapted from Clark, Thomas & Bao (2012). In addition to avoiding Borel’s paradox, Bayesian melding also includes all the advantages of a standard Bayesian analysis such as identifying the most likely region of the parameter space (instead of just the most likely set of parameters), returning the sets of parameter values in that region. Each set of parameters is associated with a probability, corresponding to how well it reflects the data. These probabilities are used to construct joint distributions of the parameter values, which allow one to characterize for uncertainty, which further eases the estimation of credible intervals (CI).

A set of functions was implemented in the *strandCet* package to fit the nine-parameter aHP model based on the methodology used in Sharrow (2012). The distribution of the priors of the aHP parameters is drawn with the *HP.priors* function; the default option is a uniform distribution defined by the lower and higher bounds introduced by the user. The main function of the *strandCet* package regarding this model is called *HP.mod*, which runs all the necessary functions to fit the aHP in one step via Bayesian Melding with IMIS and optimization using the following functions implemented in the package (in this order): *loop.optim* (performs the optimizer step in the IMIS procedure), *samp.postopt* (samples the parameters from each run of the optimizer step in which the likelihood for that run exceeds the maximum likelihood from the prior), *like.resamp* (defines some necessary arguments and removes missing values from the matrices generated) and *final.resamp* (performs the final re-sampling step in the Bayesian Melding with IMIS procedure for the nine aHP parameters).

The *HP.mod* function performs 10 default IMIS iterations and 10 optimizer iterations to sample 500 sets of parameter values in the final resample from the posterior distribution. The algorithm ends when the importance sampling weights are reasonably uniform (i.e., the expected fraction of unique points in the resample is at least 1−1/*e* = 0.632) (Raftery & Bao, 2010). The median and 90% CI of the nine parameters of the aHP model are estimated with this method. The 90% CI of the natural, total and anthropogenic mortality rates can be also estimated using the *HP.CI* function. Note that all these parameters can be defined by the user.

### Leslie matrix

Discrete age-structured models based on Leslie matrices are used to project populations over time. The annual abundance in a Leslie model (*N_t_*_+1_) is based on the multiplication of a matrix *A* by a vector representing the population structure at a given time *N_t_* defined by Eq. (6).

(6) $${N_{t + 1}} = A*{N_t}$$

Matrix *A*, in Eq. (7), is composed of an expression of fecundity by age class *F* (i.e., average number of calves born at the end of a time unit and the number of mothers in each age class at the end of the previous interval) and a survival probability during the time interval at each age *P*. The model uses only fecundity of females assuming that the number of mature males is not a limiting factor in the reproductive output of the population.

(7) $$A = \left[ {\matrix{ {{F_0}} & {{F_1}} & \cdots & {{F_{n-1}}} & {{F_n}} \cr {{P_0}} & 0 & \cdots & 0 & 0 \cr 0 & {{P_1}} & \cdots & 0 & 0 \cr \vdots & \vdots & \ldots & \vdots & \vdots \cr \vdots & \vdots & \ldots & \vdots & \vdots \cr 0 & 0 & 0 & {{P_{n-1}}} & 0 \cr } } \right]$$

The function *Leslie.matrix* implemented in the *strandCet* package generates a demographic matrix from the age-specific cumulative survival rates and the fertility-at-age (i.e., pregnancy rate) of females. The sex ratio at birth can be set by the user. The trajectory of the theoretical population generated with the Leslie matrix can be projected using the *Leslie.pred* function a given number of years set by the user. By default, 100 years is used which is commonly considered as an adequately long-time period, but other criteria as the three-generation time proposed by the International Union for Conservation of Nature can be used.

In order to measure the development and characteristics of the projected population a function (*eigen.analysis*) was developed following Lotka (1939) to calculate the annual growth of the population (i.e., percentage by which the population increases or decreases annually), the net production of the population (i.e., number of female calves that an average female produces) and the generation time (i.e., average age of reproductively active females).

### Example

To demonstrate the applicability of the methodology described and the functionality of the package, a case study with all steps needed to carry out the analysis are presented in the Supplemental Information, also accessible in https://rpubs.com/CSaavedra/strandCet_example (see also examples of real data included in the package manual). The data used was published by Stolen & Barlow (2003) and corresponds to 220 aged bottlenose dolphins (*Tursiops truncatus*) stranded in the Indian River Lagoon System (Florida, USA). Evidences suggest that this population is resident to the river system and not susceptible to any forms of anthropogenic mortality (e.g., bycatch). The main functions used in the analysis are described in Table 1 (see the Supplemental Information for the analysis details).

**Table 1:** Main functions of the package *strandCet* used in the example analysis.

Function	Description
life.tab	Constructs a regular life table
Si.mod	Performs a Siler model
Si.pred	Predicts the Siler mortality components
Est.life.tab	Estimates a life table with the Siler mortality estimates
HP.priors	Compiles priors in the format required by the adapted Heligman-Pollard model
HP.mod	Performs an adapted Heligman-Pollard model
HP.pred	Predicts the adapted Heligman-Pollard mortality components
HP.CI	Calculates credible intervals for the Heligman-Pollard mortality components
life.Leslie	Constructs the life table required for constructing a Leslie matrix
Leslie.matrix	Constructs Leslie matrices
Leslie.pred	Projects Leslie matrices
eigen.analysis	Calculates population parameters from the Leslie projections

**Figure 1:**
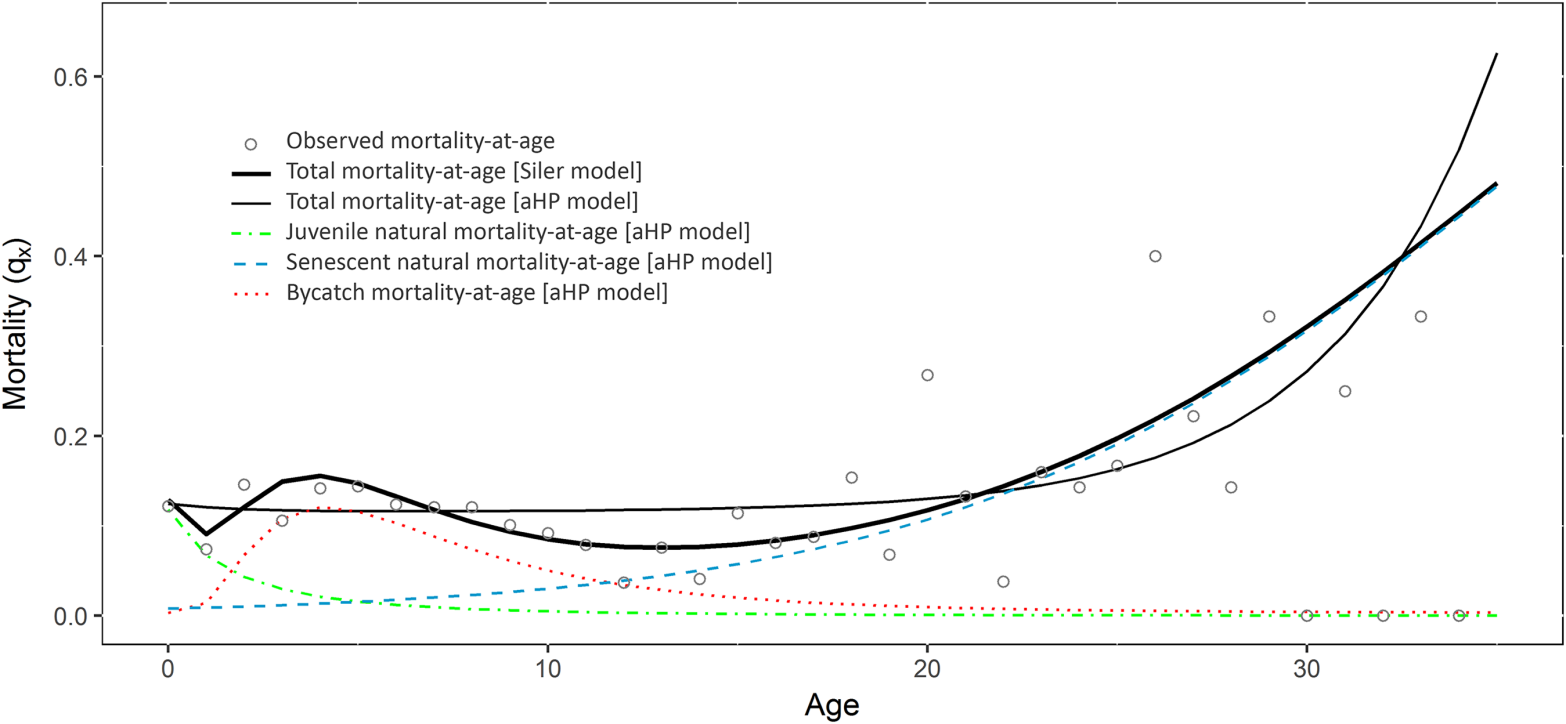
Mortality at age estimated fitting the Siler and the adapted Heligman-Pollard (aHP) models to the data with bycatch. Observed mortality-at-age (circles). Total mortality-at-age modelled with the Siler (black thin line) and the aHP (black thick line) models. Juvenile natural mortality-at-age (blue dashed line) estimated with the aHP. Senescent natural mortality-at-age (green dot-dashed line) estimated with the aHP. Bycatch mortality-at-age (red dotted line) estimated with the aHP.

A Siler model was fitted to the above-described data in order to calculate total mortality-at-age. An example of how to remove age classes before fitting the model is also included. Then, a theoretical sample of normally distributed bycaught dolphins (with mode before maturity) was added to the original data. The total, natural and bycatch mortality-at-age of this new dataset were estimated using the aHP model. Figure 1 displays the aHP model components (i.e., juvenile and senescent natural mortality, and bycatch mortality), the sum of all three (total mortality) and the total mortality estimated with the Siler model. Two Leslie matrices were projected (i.e., with natural and total mortality estimated with the aHP model), and population parameters calculated (see results in Supplemental Information or in https://rpubs.com/CSaavedra/strandCet_example).

## Discussion

The determination of marine mammal mortality is often impaired due to the lack of direct and unbiased data, especially in the case of cetaceans. Data from strandings are probably the best source of information for demographic studies for many cetaceans and the only source available for most of them. Other sources of information, such as the widely used photo-ID for the identification of individual animals, can be very reliable but only allows the user to calculate total mortality for coastal populations and species with medium or high degree of residence (Wells & Scott, 1990; Jefferson et al., 2012).

The use of the Siler model to estimate mortality-at-age from stranded cetaceans enables one to estimate only total mortality (Stolen & Barlow, 2003; Mannocci et al., 2012), which reduces its utility in populations with external causes of mortality such as bycatch. Some studies have addressed this limitation by combining strandings with catch data in fisheries to estimate natural mortality (Secchi & Fletcher, 2004; Moore & Read, 2008). However, this requires a fishery monitoring program which many fisheries lack.

In this study, a methodology (based on an aHP) that only requires the use of stranding data to estimate the total, natural, and bycatch mortality-at-age of a species or population is proposed. The *strandCet* package includes all functions needed to apply this methodology using Bayesian methods for fitting the aHP model, which allows the user to set non-informative priors, and also to apply constraints using information coming from other sources.

It should be noted that the method assumes that the age structure is in equilibrium, which would not be the case if the population is growing or diminishing. Most demographic analyses of long-lived, data-deficient species employ equilibrium models (Moore & Read, 2008). Whilst this limiting caveat can be avoided through the use of dynamic models (see Saavedra, 2017), which require considerably more data than in is usually available. However, demographic estimates for species with high survival rates and long generation times may be reasonably robust to stochastic fluctuation in age stability (see Stolen & Barlow, 2003) and the use of long time series of data and smooth functions such as those applied in the aHP model may help buffer against temporary effects on age-specific survival. Unrealistic fits may be obtained for data with unconventional patterns due to the model’s functional form. Similarly, there is a danger of over fitting when estimating the nine model parameters from observations including relatively small numbers of ages. Fortunately, both these issues can usually be resolved through the use of informative priors (Dellaportas, Smith & Stavropoulos, 2001). Future versions may include new utilities to address these issues, and others such as plotting capabilities, goodness-of-fit tests and model selection functionalities.

The Leslie model implemented in the *strandCet* package allows the user to use the aHP outputs to explore the future size and age composition of a population, and how they may change under different scenarios. In the proposed example two Leslie matrices were projected and population growth rates were calculated for populations with and without bycatch. This enables researchers and managers to establish bycatch limit reference points to avoid decreasing of populations. However, because of the use of static reproductive parameters, this approach does not take into account possible density dependence phenomena that affect most natural populations. This should be taken into account when populations grow or decrease in a long-term sustained manner. The mortality-at-age calculated with this model (both bycatch and natural mortality) can also be used as input to more complex dynamic models (e.g., GADGET see Saavedra, 2017 and Begley & Howell, 2004).

## Conclusions

The methodology developed represents a step forward in this field allowing the exploration of population trajectories under different scenarios of anthropogenic mortality and therefore the establishment of the maximum limits of (anthropogenic) mortality that populations can withstand. The R package provides a quick and user-friendly tool to explore the consequences of management decisions.

## Supplemental Information

10.7717/peerj.5768/supp-1 Supplemental Information 1 Markdown document (viewable in the browser) with the example analysis using the *strandCet* package.

10.7717/peerj.5768/supp-2 Supplemental Information 2 Script (for using in R) with the example analysis using the *strandCet* package.

## Additional Information and Declarations

### Data Availability

The following information was supplied regarding data availability: strandCet: Estimation of Biological Parameters from Stranded Cetaceans: https://CRAN.R-project.org/package=strandCet; GitHub, strandCet: http://github.com/milokmilo/strandCet; GitHub, strandCet_example: https://github.com/milokmilo/strandCet_example; RPubs, strandCet_example: https://rpubs.com/CSaavedra/strandCet_example.
