# Supplementary material for: strandCet: R package for estimating natural and non-natural mortality-at-age of cetaceans from age-structured strandings
Source: PeerJ. 2018 Oct 9;6:e5768. doi: 10.7717/peerj.5768 (PMC6183513; doi:10.7717/peerj.5768)
Supplement: Supplemental Information 1 [file peerj-06-5768-s001.html]

strandCet: R package for estimating natural and anthropogenic mortality-at-age of cetaceans from age-structured strandings.


# *strandCet*: R package for estimating natural and anthropogenic mortality-at-age of cetaceans from age-structured strandings.

#### *Camilo Saavedra*

## Example

##### In this document can be found the code used to run the examples of the paper titled “*strandCet*: R package for estimating natural and anthropogenic mortality-at-age of cetaceans from age-structured strandings”. It should be noted that model fits and estimated parameters could vary slightly from one run to another. General stepwise instructions for the different functions and their options, as well as other examples can be found in the *strandCet* manual https://cran.r-project.org/web/packages/strandCet/strandCet.pdf. For further details see the package help files > help(package = “strandCet”).

#### Load necesary packages.

##### Note that these packages must be previously installed.

```
library(strandCet)
library(ggplot2)
```

### Original data.

##### From Stolen and Barlow (2003).

```
N <- c(36,14,32,15,13,8,4,5,3,3,2,3,3,6,3,8,5,5,8,3,11,4,1,4,3,3,6,2,1,2,0,1,0,1,0,2)
age <- 0:35
data.A <- data.frame(age, N)
```

#### Life table from observed data (original data without bycatch).

```
life.A <- life.tab(data.A)
```

**Table 1: Life table performed using the original data without bycach.** Age at the beginning of the interval (age). Number of observed deaths at age x (Mx). Number of survivors at age x (Sx). Number of survivors at age x in a theoretical cohort starting with n individuals (nx). Number of deaths at age x in a theoretical cohort starting with n individuals (dx). Probability of death between ages x and x + n (qx). Probability of survival to exact age x (lx). Life expectancy at age x (ex). Instant death rate at age x (Zx).

```
##  age Mx  Sx       nx      dx    qx    lx     ex    Zx
##    0 36 220 1000.000 163.636 0.164 1.000 10.159 0.179
##    1 14 184  836.364  63.636 0.076 0.836 10.951 0.079
##    2 32 170  772.727 145.455 0.188 0.773 10.771 0.209
##    3 15 138  627.273  68.182 0.109 0.627 12.036 0.115
##    4 13 123  559.091  59.091 0.106 0.559 12.382 0.112
##    5  8 110  500.000  36.364 0.073 0.500 12.727 0.076
##    6  4 102  463.636  18.182 0.039 0.464 12.647 0.040
##    7  5  98  445.455  22.727 0.051 0.445 12.122 0.052
##    8  3  93  422.727  13.636 0.032 0.423 11.720 0.033
##    9  3  90  409.091  13.636 0.033 0.409 11.078 0.034
##   10  2  87  395.455   9.091 0.023 0.395 10.425 0.023
##   11  3  85  386.364  13.636 0.035 0.386  9.647 0.036
##   12  3  82  372.727  13.636 0.037 0.373  8.963 0.037
##   13  6  79  359.091  27.273 0.076 0.359  8.266 0.079
##   14  3  73  331.818  13.636 0.041 0.332  7.863 0.042
##   15  8  70  318.182  36.364 0.114 0.318  7.157 0.121
##   16  5  62  281.818  22.727 0.081 0.282  6.952 0.084
##   17  5  57  259.091  22.727 0.088 0.259  6.474 0.092
##   18  8  52  236.364  36.364 0.154 0.236  6.000 0.167
##   19  3  44  200.000  13.636 0.068 0.200  5.909 0.071
##   20 11  41  186.364  50.000 0.268 0.186  5.268 0.312
##   21  4  30  136.364  18.182 0.133 0.136  5.833 0.143
##   22  1  26  118.182   4.545 0.038 0.118  5.577 0.039
##   23  4  25  113.636  18.182 0.160 0.114  4.760 0.174
##   24  3  21   95.455  13.636 0.143 0.095  4.476 0.154
##   25  3  18   81.818  13.636 0.167 0.082  4.056 0.182
##   26  6  15   68.182  27.273 0.400 0.068  3.667 0.511
##   27  2   9   40.909   9.091 0.222 0.041  4.444 0.251
##   28  1   7   31.818   4.545 0.143 0.032  4.429 0.154
##   29  2   6   27.273   9.091 0.333 0.027  4.000 0.405
##   30  0   4   18.182   0.000 0.000 0.018  4.500 0.000
##   31  1   4   18.182   4.545 0.250 0.018  3.500 0.288
##   32  0   3   13.636   0.000 0.000 0.014  3.333 0.000
##   33  1   3   13.636   4.545 0.333 0.014  2.333 0.405
##   34  0   2    9.091   0.000 0.000 0.009  2.000 0.000
##   35  2   2    9.091   9.091 1.000 0.009  1.000 1.000
```

### Data with the first age class underrepresented.

##### Stolen and Barlow (2003) considered that the number of stranded cetaceans are not negatively biased in the first age classes.

##### Only one third of the strandings of calves (first age class) were considered in this dataset to simulate a scenario with underrepresentation of calves.

```
Nyoung <- c(N[c(1)]/3, N[-c(1)])
data.B <- data.frame(age, Nyoung)
```

#### Life table from observed data (data with the first age class underrepresented).

```
life.B <- life.tab(data.B)
```

**Table 2: Life table performed using the data with the first age class underrepresented.** Age at the beginning of the interval (age). Number of observed deaths at age x (Mx). Number of survivors at age x (Sx). Number of survivors at age x in a theoretical cohort starting with n individuals (nx). Number of deaths at age x in a theoretical cohort starting with n individuals (dx). Probability of death between ages x and x + n (qx). Probability of survival to exact age x (lx). Life expectancy at age x (ex). Instant death rate at age x (Zx).

```
##  age Mx  Sx       nx      dx    qx    lx     ex    Zx
##    0 12 196 1000.000  61.224 0.061 1.000 11.281 0.063
##    1 14 184  938.776  71.429 0.076 0.939 10.951 0.079
##    2 32 170  867.347 163.265 0.188 0.867 10.771 0.209
##    3 15 138  704.082  76.531 0.109 0.704 12.036 0.115
##    4 13 123  627.551  66.327 0.106 0.628 12.382 0.112
##    5  8 110  561.224  40.816 0.073 0.561 12.727 0.076
##    6  4 102  520.408  20.408 0.039 0.520 12.647 0.040
##    7  5  98  500.000  25.510 0.051 0.500 12.122 0.052
##    8  3  93  474.490  15.306 0.032 0.474 11.720 0.033
##    9  3  90  459.184  15.306 0.033 0.459 11.078 0.034
##   10  2  87  443.878  10.204 0.023 0.444 10.425 0.023
##   11  3  85  433.673  15.306 0.035 0.434  9.647 0.036
##   12  3  82  418.367  15.306 0.037 0.418  8.963 0.037
##   13  6  79  403.061  30.612 0.076 0.403  8.266 0.079
##   14  3  73  372.449  15.306 0.041 0.372  7.863 0.042
##   15  8  70  357.143  40.816 0.114 0.357  7.157 0.121
##   16  5  62  316.327  25.510 0.081 0.316  6.952 0.084
##   17  5  57  290.816  25.510 0.088 0.291  6.474 0.092
##   18  8  52  265.306  40.816 0.154 0.265  6.000 0.167
##   19  3  44  224.490  15.306 0.068 0.224  5.909 0.071
##   20 11  41  209.184  56.122 0.268 0.209  5.268 0.312
##   21  4  30  153.061  20.408 0.133 0.153  5.833 0.143
##   22  1  26  132.653   5.102 0.038 0.133  5.577 0.039
##   23  4  25  127.551  20.408 0.160 0.128  4.760 0.174
##   24  3  21  107.143  15.306 0.143 0.107  4.476 0.154
##   25  3  18   91.837  15.306 0.167 0.092  4.056 0.182
##   26  6  15   76.531  30.612 0.400 0.077  3.667 0.511
##   27  2   9   45.918  10.204 0.222 0.046  4.444 0.251
##   28  1   7   35.714   5.102 0.143 0.036  4.429 0.154
##   29  2   6   30.612  10.204 0.333 0.031  4.000 0.405
##   30  0   4   20.408   0.000 0.000 0.020  4.500 0.000
##   31  1   4   20.408   5.102 0.250 0.020  3.500 0.288
##   32  0   3   15.306   0.000 0.000 0.015  3.333 0.000
##   33  1   3   15.306   5.102 0.333 0.015  2.333 0.405
##   34  0   2   10.204   0.000 0.000 0.010  2.000 0.000
##   35  2   2   10.204  10.204 1.000 0.010  1.000 1.000
```

### Data with theoretical bycaught dolphins added.

##### Stolen and Barlow (2003) considered that the stranded cetaceans belonged to a population not affected by direct anthropogenic mortality.

##### Bycaught dolphins were added following a gaussian distribution to simulate a population affected by incidental catch

```
set.seed(123)
Nbyc <- N + c(sort(round(rnorm(6, mean = 10, sd = 5))),
              rev(sort(round(rnorm(6, mean = 10, sd = 5)))), rep(0,24))
data.C <- data.frame(age, Nbyc)
```

#### Life table from observed data (data with theoretical bycaught dolphins).

```
life.C <- life.tab(data.C)
```

**Table 3: Life table performed using the data with theoretical bycaught dolphins.** Age at the beginning of the interval (age). Number of observed deaths at age x (Mx). Number of survivors at age x (Sx). Number of survivors at age x in a theoretical cohort starting with n individuals (nx). Number of deaths at age x in a theoretical cohort starting with n individuals (dx). Probability of death between ages x and x + n (qx). Probability of survival to exact age x (lx). Life expectancy at age x (ex). Instant death rate at age x (Zx).

```
##  age Mx  Sx       nx      dx    qx    lx    ex    Zx
##    0 43 353 1000.000 121.813 0.122 1.000 8.666 0.130
##    1 23 310  878.187  65.156 0.074 0.878 8.729 0.077
##    2 42 287  813.031 118.980 0.146 0.813 8.348 0.158
##    3 26 245  694.051  73.654 0.106 0.694 8.608 0.112
##    4 31 219  620.397  87.819 0.142 0.620 8.511 0.153
##    5 27 188  532.578  76.487 0.144 0.533 8.750 0.155
##    6 20 161  456.091  56.657 0.124 0.456 9.050 0.133
##    7 17 141  399.433  48.159 0.121 0.399 9.191 0.128
##    8 15 124  351.275  42.493 0.121 0.351 9.315 0.129
##    9 11 109  308.782  31.161 0.101 0.309 9.459 0.106
##   10  9  98  277.620  25.496 0.092 0.278 9.408 0.096
##   11  7  89  252.125  19.830 0.079 0.252 9.258 0.082
##   12  3  82  232.295   8.499 0.037 0.232 8.963 0.037
##   13  6  79  223.796  16.997 0.076 0.224 8.266 0.079
##   14  3  73  206.799   8.499 0.041 0.207 7.863 0.042
##   15  8  70  198.300  22.663 0.114 0.198 7.157 0.121
##   16  5  62  175.637  14.164 0.081 0.176 6.952 0.084
##   17  5  57  161.473  14.164 0.088 0.161 6.474 0.092
##   18  8  52  147.309  22.663 0.154 0.147 6.000 0.167
##   19  3  44  124.646   8.499 0.068 0.125 5.909 0.071
##   20 11  41  116.147  31.161 0.268 0.116 5.268 0.312
##   21  4  30   84.986  11.331 0.133 0.085 5.833 0.143
##   22  1  26   73.654   2.833 0.038 0.074 5.577 0.039
##   23  4  25   70.822  11.331 0.160 0.071 4.760 0.174
##   24  3  21   59.490   8.499 0.143 0.059 4.476 0.154
##   25  3  18   50.992   8.499 0.167 0.051 4.056 0.182
##   26  6  15   42.493  16.997 0.400 0.042 3.667 0.511
##   27  2   9   25.496   5.666 0.222 0.025 4.444 0.251
##   28  1   7   19.830   2.833 0.143 0.020 4.429 0.154
##   29  2   6   16.997   5.666 0.333 0.017 4.000 0.405
##   30  0   4   11.331   0.000 0.000 0.011 4.500 0.000
##   31  1   4   11.331   2.833 0.250 0.011 3.500 0.288
##   32  0   3    8.499   0.000 0.000 0.008 3.333 0.000
##   33  1   3    8.499   2.833 0.333 0.008 2.333 0.405
##   34  0   2    5.666   0.000 0.000 0.006 2.000 0.000
##   35  2   2    5.666   5.666 1.000 0.006 1.000 1.000
```

### Siler Model (original data without bycatch).

##### In the simplest scenario, there are no negative biases in the number of stranded animals of the first age classes and we are interested in calculating only the total mortality (since it is considered that the population is not affected by major anthropogenic pressures).

#### Siler model (original data without bycatch).

```
modSI <-  Si.mod(data.A)
```

#### Predict the Siler model (original data without bycatch).

```
predSI <- Si.pred(data.A, modSI)
```

#### Life table of the Siler model (original data without bycatch).

```
life.Siler <- Est.life.tab(Est.qx = predSI$qx.tot, age = 0:35, n = 1000)
```

**Table 4: Life table of the original data without bycach modelled using the Siler model.** Age at the beginning of the interval (Age). Probability of death between ages x and x + n (qx). Number of survivors at age x in a theoretical cohort starting with n individuals (nx). Number of deaths at age x in a theoretical cohort starting with n individuals (dx). Probability of survival to exact age x (lx). Life expectancy at age x (ex).Instant death rate at age x (Zx).

```
##  age    qx       nx      dx    lx     ex    Zx
##    0 0.208 1000.000 207.577 1.000  9.089 0.233
##    1 0.170  792.423 134.665 0.792 10.209 0.186
##    2 0.139  657.758  91.158 0.658 11.094 0.149
##    3 0.113  566.600  63.901 0.567 11.718 0.120
##    4 0.092  502.699  46.182 0.503 12.080 0.096
##    5 0.075  456.517  34.374 0.457 12.201 0.078
##    6 0.063  422.144  26.416 0.422 12.113 0.065
##    7 0.053  395.728  21.086 0.396 11.855 0.055
##    8 0.047  374.642  17.629 0.375 11.466 0.048
##    9 0.044  357.013  15.557 0.357 10.983 0.045
##   10 0.043  341.456  14.535 0.341 10.438 0.043
##   11 0.044  326.921  14.318 0.327  9.857 0.045
##   12 0.047  312.604  14.710 0.313  9.263 0.048
##   13 0.052  297.894  15.543 0.298  8.671 0.054
##   14 0.059  282.351  16.660 0.282  8.093 0.061
##   15 0.067  265.691  17.912 0.266  7.538 0.070
##   16 0.077  247.779  19.154 0.248  7.010 0.080
##   17 0.089  228.624  20.251 0.229  6.514 0.093
##   18 0.101  208.373  21.079 0.208  6.050 0.107
##   19 0.115  187.294  21.538 0.187  5.618 0.122
##   20 0.130  165.756  21.553 0.166  5.218 0.139
##   21 0.146  144.204  21.085 0.144  4.849 0.158
##   22 0.164  123.119  20.135 0.123  4.508 0.179
##   23 0.182  102.984  18.740 0.103  4.194 0.201
##   24 0.201   84.244  16.975 0.084  3.904 0.225
##   25 0.222   67.269  14.941 0.067  3.637 0.251
##   26 0.244   52.328  12.758 0.052  3.390 0.279
##   27 0.267   39.570  10.549 0.040  3.161 0.310
##   28 0.290   29.021   8.430 0.029  2.946 0.343
##   29 0.315   20.591   6.496 0.021  2.743 0.379
##   30 0.342   14.095   4.815 0.014  2.546 0.418
##   31 0.369    9.280   3.423 0.009  2.348 0.460
##   32 0.397    5.857   2.327 0.006  2.135 0.506
##   33 0.427    3.530   1.507 0.004  1.884 0.557
##   34 0.458    2.023   0.926 0.002  1.542 0.612
##   35 0.490    1.096   0.537 0.001  1.000 1.000
```

#### Siler plot (original data without bycatch).

```
Sicurves <- ggplot(predSI, aes(age, qx.tot)) +
  geom_line(colour = "red", lty = 1, size = 0.5) +
  geom_point(data = life.A, aes(age, qx), shape = 1, colour = "grey50") +
  ylim(0, 0.5) +
  ylab(expression("Mortality (q" [x]* ")")) + xlab("Age") +
  ggtitle("") +
  theme(panel.background =  element_rect(fill = NA, colour = "black", size = 0.5), 
        legend.title = element_blank(), legend.position = "none")
```

**Fig. 1: Mortality-at-age estimated fitting the Siler model to the original data without bycatch**. Observed mortality rates (circles) and total mortality-at-age modelled with the Siler model (red line).

### Siler Model (data with the first age class underrepresented).

##### The first age classes are usually underrepresented in strandings. One option is to remove the ages considered negatively biased fitting the model to the remaining ages. If this bias/underrepresentation is ignored, we can get wrong fits, even with lower mortality rates in the first age classes than in the juvenile ages.

#### Siler model (data with the first age class underrepresented).

```
modSI.R <-  Si.mod(data.B, rm = 1)
modSI <-  Si.mod(data.B)
```

#### Predict the Siler model (data with the first age class underrepresented).

```
predSI.R <- Si.pred(data.B, modSI.R, rm = 1)
predSI <- Si.pred(data.B, modSI)
```

#### Life table of the Siler model (data with the first age class underrepresented).

```
life.Siler <- Est.life.tab(Est.qx = predSI.R$qx.tot, age = 0:35, n = 1000)
```

**Table 5: Life table of the data with the first age class underrepresented modelled using the Siler model.** Age at the beginning of the interval (Age). Probability of death between ages x and x + n (qx). Number of survivors at age x in a theoretical cohort starting with n individuals (nx). Number of deaths at age x in a theoretical cohort starting with n individuals (dx). Probability of survival to exact age x (lx). Life expectancy at age x (ex).Instant death rate at age x (Zx).

```
##  age    qx       nx      dx    lx     ex    Zx
##    0 0.218 1000.000 217.828 1.000  8.880 0.246
##    1 0.177  782.172 138.196 0.782 10.075 0.194
##    2 0.143  643.976  91.919 0.644 11.023 0.154
##    3 0.115  552.057  63.521 0.552 11.691 0.122
##    4 0.093  488.535  45.365 0.489 12.082 0.097
##    5 0.075  443.170  33.433 0.443 12.216 0.078
##    6 0.062  409.737  25.492 0.410 12.131 0.064
##    7 0.053  384.245  20.240 0.384 11.870 0.054
##    8 0.046  364.005  16.885 0.364 11.474 0.047
##    9 0.043  347.120  14.918 0.347 10.984 0.044
##   10 0.042  332.202  13.993 0.332 10.432 0.043
##   11 0.044  318.209  13.859 0.318  9.847 0.045
##   12 0.047  304.350  14.316 0.304  9.249 0.048
##   13 0.052  290.034  15.192 0.290  8.657 0.054
##   14 0.059  274.842  16.327 0.275  8.080 0.061
##   15 0.068  258.515  17.574 0.259  7.527 0.070
##   16 0.078  240.941  18.789 0.241  7.003 0.081
##   17 0.089  222.152  19.841 0.222  6.511 0.094
##   18 0.102  202.310  20.614 0.202  6.051 0.107
##   19 0.116  181.697  21.013 0.182  5.624 0.123
##   20 0.131  160.683  20.975 0.161  5.229 0.140
##   21 0.147  139.708  20.468 0.140  4.864 0.158
##   22 0.164  119.240  19.499 0.119  4.527 0.179
##   23 0.182   99.740  18.111 0.100  4.217 0.200
##   24 0.201   81.629  16.379 0.082  3.931 0.224
##   25 0.221   65.250  14.402 0.065  3.666 0.249
##   26 0.242   50.849  12.294 0.051  3.421 0.277
##   27 0.264   38.554  10.173 0.039  3.194 0.306
##   28 0.287   28.381   8.144 0.028  2.980 0.338
##   29 0.311   20.238   6.295 0.020  2.777 0.373
##   30 0.336   13.943   4.687 0.014  2.579 0.410
##   31 0.362    9.255   3.354 0.009  2.378 0.450
##   32 0.390    5.901   2.300 0.006  2.162 0.494
##   33 0.418    3.602   1.506 0.004  1.903 0.541
##   34 0.448    2.096   0.938 0.002  1.552 0.593
##   35 0.478    1.158   0.554 0.001  1.000 1.000
```

#### Siler plot (data with the first age class underrepresented).

```
Sicurves <- ggplot(predSI.R, aes(age, qx.tot)) +
  geom_line(colour = "red", lty = 1, size = 0.5) +
  geom_line(data = predSI, colour = "black", lty = 2, size = 0.5) +
  geom_point(data = life.B, aes(age, qx), shape = 1, colour = "grey50") +
  geom_point(data = life.B[c(1),], aes(age, qx), shape = 16, colour="grey50") +
  ylim(0, 0.5) +
  ylab(expression("Mortality (q" [x]* ")")) + xlab("Age") +
  ggtitle("") +
  theme(panel.background =  element_rect(fill = NA, colour = "black", size = 0.5), 
        legend.title = element_blank(), legend.position = "none")
```

**Fig. 2: Mortality-at-age estimated fitting the Siler model to the data with the first age class underrepresented**. Observed mortality rates used (circles) and not used (dot) for fitting the model. Total mortality-at-age modelled removing the first age class (red line) and mortality-at-age modelled using all ages (dashed line).

### Heligman-Pollard Model (data with theoretical bycaught dolphins).

##### Mortality-at-age of a population affected by high levels of bycatch (which mainly affects young ages) should not be fitted with a Siler model. Mortality rates may not show a tipical U-shape, so the fit could not be adequate. In addition, it is only posible to calculate the total mortality. In these cases, the adapted Heligman-Pollard model is more appropriate.

#### Siler model (data with theoretical bycaught dolphins).

```
modSI <-  Si.mod(data.C)
```

#### Predict the Siler model (data with theoretical bycaught dolphins).

```
predSI <- Si.pred(data.C, modSI)
```

#### Select non-informative priors for the Heligman-Pollard model (data with theoretical bycaught dolphins).

```
priors <- data.frame(priors.lo = c(0,0,0,0,0,0,1,0,1),
                     priors.hi = c(1,10,1,0.01,0.5,10,15,0.01,1.5))
```

#### Compile priors in the required format (data with theoretical bycaught dolphins).

```
q0 <- HP.priors(pri.lo = priors$priors.lo,
                pri.hi = priors$priors.hi,
                theta.dim = 9)
```

#### Run the Heligman-Pollard model (data with theoretical bycaught dolphins).

**Table 6: Heligman-Pollard parameters.** Parameters A, B and C represent the juvenile mortality. D, E, F and I represent the “accident hump”. G and H represent the senescent mortality.

```
##   Low CI Median High CI
## A  0.058  0.158   0.257
## B  0.309  1.397   2.531
## C  0.328  0.436   0.527
## I  0.001  0.003   0.004
## D  0.099  0.118   0.135
## E  0.871  1.160   1.427
## F  3.625  4.121   4.697
## G  0.007  0.008   0.009
## H  1.135  1.145   1.153
```

#### Predict the Heligman-Pollard model (data with theoretical bycaught dolphins).

```
predHP <- HP.pred(life = life.C, HPout = modHP, age = age)
```

**Table 7: Life table of the data with theoretical bycaught dolphins modelled using the adapted Heligman-Pollard model.** Age at the beginning of the interval (age). Number of observed deaths at age x (Mx). Total probability of death between ages x and x + n (qx.tot). Natural probability of death between ages x and x + n (qx.nat). Young probability of death between ages x and x + n (qx.young). Probability of death due to an externl risk between ages x and x + n (qx.risk). Adult or senescent probability of death between ages x and x + n (qx.adult).

```
##  age Mx     qx.tot     qx.nat     qx.young     qx.risk    qx.adult
##    0 43 0.12921491 0.12621491 0.1182783993 0.003000000 0.007936508
##    1 23 0.09072135 0.07619651 0.0671196496 0.014524841 0.009076856
##    2 42 0.12080966 0.05345528 0.0430759386 0.067354385 0.010379339
##    3 26 0.14946720 0.04148808 0.0296215977 0.107979121 0.011866485
##    4 31 0.15576132 0.03488282 0.0213190363 0.120878496 0.013563786
##    5 27 0.14734892 0.03135631 0.0158562562 0.115992618 0.015500051
##    6 20 0.13298221 0.02980088 0.0120931241 0.103181322 0.017707760
##    7 17 0.11783829 0.02963254 0.0094090769 0.088205750 0.020223459
##    8 15 0.10435747 0.03052959 0.0074414332 0.073827881 0.023088156
##    9 11 0.09345666 0.03231418 0.0059664450 0.061142483 0.026347733
##   10  9 0.08531525 0.03489339 0.0048400550 0.050421862 0.030053337
##   11  7 0.07979997 0.03822801 0.0039662418 0.041571965 0.034261768
##   12  3 0.07667549 0.04231493 0.0032791340 0.034360560 0.039035796
##   13  6 0.07570143 0.04717685 0.0027324307 0.028524581 0.044444422
##   14  3 0.07667246 0.05285591 0.0022929008 0.023816549 0.050563013
##   15  8 0.07943100 0.05940953 0.0019362581 0.020021465 0.057473277
##   16  5 0.08386755 0.06690749 0.0016444690 0.016960055 0.065263024
##   17  5 0.08991581 0.07542960 0.0014039531 0.014486209 0.074025648
##   18  8 0.09754599 0.08506360 0.0012043540 0.012482394 0.083859245
##   19  3 0.10675775 0.09590300 0.0010376846 0.010854752 0.094865313
##   20 11 0.11757325 0.10804465 0.0008977216 0.009528604 0.107146927
##   21  4 0.13003054 0.12158592 0.0007795710 0.008444619 0.120806347
##   22  1 0.14417698 0.13662133 0.0006793512 0.007555645 0.135941982
##   23  4 0.16006283 0.15323866 0.0005939597 0.006824168 0.152644700
##   24  3 0.17773468 0.17151440 0.0005208986 0.006220283 0.170993500
##   25  3 0.19722888 0.19150878 0.0004581437 0.005720094 0.191050637
##   26  6 0.21856486 0.21326040 0.0004040445 0.005304453 0.212856360
##   27  2 0.24173874 0.23678076 0.0003572473 0.004957974 0.236423516
##   28  1 0.26671726 0.26204901 0.0003166360 0.004668252 0.261732371
##   29  2 0.29343261 0.28900735 0.0002812858 0.004425260 0.288726066
##   30  0 0.32177848 0.31755762 0.0002504267 0.004220861 0.317307192
##   31  1 0.35160781 0.34755938 0.0002234149 0.004048434 0.347335965
##   32  0 0.38273273 0.37883015 0.0001997098 0.003902575 0.378630445
##   33  1 0.41492679 0.41114793 0.0001788555 0.003778858 0.410969075
##   34  0 0.44792981 0.44425617 0.0001604665 0.003673648 0.444095700
##   35  2 0.48145506 0.47787111 0.0001442154 0.003583950 0.477726890
```

#### Heligman-Pollard plot (data with theoretical bycaught dolphins).

```
HPcurves <- ggplot(predHP, aes(age, qx.tot)) +
    geom_line(colour = "black", lty=1, size = 0.8) +
    geom_line(data = predSI, colour = "black", lty=1, size = 0.5) +
    geom_line(aes(age, qx.young), colour = "green", lty = 4) +
    geom_line(aes(age, qx.risk), colour = "red", lty = 3, size = 0.5) +
    geom_line(aes(age, qx.adult), colour = "deepskyblue3", lty = 2) +
    geom_point(data = life.C, aes(age, qx), shape=1, colour="grey50") +
    ylim(0,0.65) +
    ylab(expression("Mortality (q" [x]* ")")) + xlab("Age") +
    ggtitle("") +
    theme(panel.background =  element_rect(fill = NA, colour = "black", size = 0.5), 
          legend.title = element_blank(), legend.position = "none")
```

\*\*Fig. 3: Mortality-at-age estimated fitting the Siler and the adapted Heligman-Pollard (aHP) models to the data with bycatch. Observed mortality-at-age (circles). Total mortality-at-age modelled with the Siler (black thin line) and the aHP (black thick line) models. Juvenile natural mortality-at-age (blue dashed line) estimated with the aHP. Senescent natural mortality-at-age (green dot-dashed line) estimated with the aHP. Bycatch mortality-at-age (red dotted line) estimated with the aHP.

### Leslie Matrices (data with theoretical bycaught dolphins).

##### Three generation time was used for projections following IUCN recommendations. Since the generation time of the bottlenose dolphins (*Tursiops truncatus*) used in this analysis is about 21 years (Taylor et al. 2007). Threfore, 65 years were used as an approximation of three generations.

#### Set a maturity vector

##### Maturity at age 7 (first parturition at age 8) with pregnancy rate of 40% (Wells et al. 1987).

```
mat <- c(0,0,0,0,0,0,0,0, rep(0.4, 28))
```

### Leslie Matrix using the total mortality estimated with the Heligman-Pollard model.

#### Get the median, low and high 90% credible intervals of the Total Heligman-Pollar mortality (using the total mortality).

```
TotalMs <- HP.CI(HPout = modHP, age = 0:35, CI = 90, M = "total")
```

#### Contruct life tables for the median and credible intervals (using the total mortality).

```
TotHP.life <- Est.life.tab(Est.qx = TotalMs$Med, age = 0:35, n = 1000)
TotHP.life.L <- Est.life.tab(Est.qx = TotalMs$Mlo, age = 0:35, n = 1000)
TotHP.life.H <- Est.life.tab(Est.qx = TotalMs$Mhi, age = 0:35, n = 1000)
```

#### Contruct Leslie life tables for the median and credible intervals (using the total mortality).

```
Tot.life <- with(TotHP.life, life.Leslie(x = 0:35, nKx = nx, nDx = dx,
                                 type = "cohort", iwidth = 1,
                                 width12 = c(1, 1)))
Tot.lifeL <- with(TotHP.life.L, life.Leslie(x = 0:35, nKx = nx, nDx = dx,
                                   type = "cohort", iwidth = 1,
                                   width12 = c(1,1)))
Tot.lifeH <- with(TotHP.life.H, life.Leslie(x = 0:35, nKx = nx, nDx = dx,
                                   type="cohort", iwidth = 1,
                                   width12  =c(1,1)))
```

#### Contruct Leslie matrices for the median and credible intervals (using the total mortality).

```
Tot.A <- Leslie.matrix(lx = Tot.life$nLx, mx = mat, infant.class = FALSE,
            one.sex = TRUE, SRB = 1, L = FALSE, peryear = 1)
Tot.AL <- Leslie.matrix(lx = Tot.lifeL$nLx, mx = mat, infant.class = FALSE,
             one.sex = TRUE, SRB = 1, L = FALSE, peryear = 1)
Tot.AH <- Leslie.matrix(lx = Tot.lifeH$nLx, mx = mat, infant.class = FALSE,
             one.sex = TRUE, SRB = 1, L = FALSE, peryear = 1)
```

#### Predict Leslie matrices (using the total mortality).

# 

```
N.tot <- Leslie.pred(A = Tot.A, no = TotHP.life$nx, tmax = 65, pop.sum = TRUE)
NL.tot <- Leslie.pred(A = Tot.AL, no = TotHP.life.L$nx, tmax = 65, pop.sum = TRUE)
NH.tot <- Leslie.pred(A = Tot.AH, no = TotHP.life.H$nx, tmax = 65, pop.sum = TRUE)
```

#### Eigenvalues (using the total mortality).

```
Tot.Aea <- eigen.analysis(Tot.A); Tot.Aea$lambda1
Tot.AeaL <- eigen.analysis(Tot.AL); Tot.AeaL$lambda1
Tot.AeaH <- eigen.analysis(Tot.AH); Tot.AeaH$lambda1
```

##### The median **population growth** (with 90% credible intervals) using the total mortality (with bycatch) fitted with the Heligman-Pollard model is 0.97 [0.95,0.98].

```
Tot.Aea$rho
Tot.AeaL$rho
Tot.AeaH$rho
```

##### The median **net production** (with 90% credible intervals) using the total mortality (with bycatch) fitted with the Heligman-Pollard model is 1.18 [1.18,1.19].

```
gen.time(Tot.A, peryear = 1)
gen.time(Tot.AL, peryear = 1)
gen.time(Tot.AH, peryear = 1)
```

##### The median **generation time** (with 90% credible intervals) using the total mortality (with bycatch) fitted with the Heligman-Pollard model is 15.49 [15.35,15.66].

### Leslie Matrix using the natural mortality estimated with the Heligman-Pollard model.

#### Get the median, low and high 90% credible intervals of the Natural Heligman-Pollar mortality (using the natural mortality only).

```
NaturalMs <- HP.CI(HPout = modHP, age = 0:35, CI = 90, M = "natural")
```

#### Contruct life tables for the median and credible intervals (using the natural mortality only).

```
NatHP.life <- Est.life.tab(Est.qx = NaturalMs$Med, age = 0:35, n = 1000)
NatHP.life.L <- Est.life.tab(Est.qx = NaturalMs$Mlo, age = 0:35, n = 1000)
NatHP.life.H <- Est.life.tab(Est.qx = NaturalMs$Mhi, age = 0:35, n = 1000)
```

#### Contruct Leslie life tables for the median and credible intervals (using the natural mortality only).

```
Nat.life <- with(NatHP.life, life.Leslie(x = age, nKx = nx, nDx = dx,
                                    type = "cohort", iwidth = 1,
                                    width12 = c(1,1)))
Nat.lifeL <- with(NatHP.life.L, life.Leslie(x = age, nKx = nx, nDx = dx,
                                      type = "cohort", iwidth = 1,
                                      width12 = c(1,1)))
Nat.lifeH <- with(NatHP.life.H, life.Leslie(x = age, nKx = nx, nDx = dx,
                                      type = "cohort", iwidth = 1,
                                      width12 = c(1,1)))
```

#### Contruct Leslie matrices for the median and credible intervals (using the natural mortality only).

```
Nat.A <- Leslie.matrix(lx = Nat.life$nLx, mx = mat, infant.class = FALSE,
            one.sex = TRUE, SRB = 1, L = FALSE, peryear = 1)
Nat.AL <- Leslie.matrix(lx = Nat.lifeL$nLx, mx = mat, infant.class = FALSE,
             one.sex = TRUE, SRB = 1, L = FALSE, peryear = 1)
Nat.AH <- Leslie.matrix(lx = Nat.lifeH$nLx, mx = mat, infant.class = FALSE,
             one.sex = TRUE, SRB = 1, L = FALSE, peryear = 1)
```

#### Predict Leslie matrices (using the natural mortality only).

```
N.nat <- Leslie.pred(A = Nat.A, no = NatHP.life$nx, tmax = 65, pop.sum = TRUE)
NL.nat <- Leslie.pred(A = Nat.AL, no = NatHP.life.L$nx, tmax = 65, pop.sum = TRUE)
NH.nat <- Leslie.pred(A = Nat.AH, no = NatHP.life.H$nx, tmax = 65, pop.sum = TRUE)
```

#### Eigenvalues (using the natural mortality only).

```
Nat.Aea <- eigen.analysis(Nat.A); Nat.Aea$lambda1
Nat.AeaL <- eigen.analysis(Nat.AL); Nat.AeaL$lambda1
Nat.AeaH <- eigen.analysis(Nat.AH); Nat.AeaH$lambda1
```

##### The median **population growth** (with 90% credible intervals) using the natural mortality (without bycatch) fitted with the Heligman-Pollard model is 1.03 [1.01,1.04].

```
Nat.Aea$rho
Nat.AeaL$rho
Nat.AeaH$rho
```

##### The median **net production** (with 90% credible intervals) using the natural mortality (without bycatch) fitted with the Heligman-Pollard model is 1.23 [1.23,1.24].

```
gen.time(Nat.A, peryear = 1)
gen.time(Nat.AL, peryear = 1)
gen.time(Nat.AH, peryear = 1)
```

##### The median **generation time** (with 90% credible intervals) using the natural mortality (without bycatch) fitted with the Heligman-Pollard model is 15.23 [15.11,15.37].

### Bibliography.

##### Stolen M., Barlow J. 2003. A model life table for bottlenose dolphins (*Tursiops truncatus*) from the Indian River Lagoon System, Florida, USA. *Marine mammal science* 19:630–649. DOI: 10.1111/j.1748-7692.2003.tb01121.x.

##### Taylor, B.L., Chivers, S.J., Larese, J., Perrin, W.F., 2007. Generation length and percent mature estimates for IUCN assessments of cetaceans. *NOAA Technical Memorandum. Southwest Fisheries Science Center.*

##### Wells, RS., Scott, MD., & Irvine, AB. (1987). *The social structure of free-ranging bottlenose dolphins*. In: HH. Genoways (Ed.), Current Mammalogy (pp. 247–305). New York: Plenum Press.
